# Minimally Invasive Chemical Biopsy Needle with Self‐Wettable Extraction Phase For In Vivo Tissue Sampling During Medical Procedures

**DOI:** 10.1002/advs.202500396

**Published:** 2025-07-22

**Authors:** Runshan Will Jiang, Wei Zhou, Marcelo Cypel, Todd L. Demmy, Gal Shafirstein, Guillermo Garza, Emily Gawrys, Joanna Bogusiewicz, Barbara Bojko, Janusz Pawliszyn

**Affiliations:** ^1^ Department of Chemistry University of Waterloo Waterloo ON N2L 3G1 Canada; ^2^ Latner Thoracic Surgery Research Laboratories Toronto General Hospital Research Institute University Health Network Toronto ON M5G 0A3 Canada; ^3^ Department of Thoracic Surgery Roswell Park Comprehensive Cancer Center Buffalo NY 14203 USA; ^4^ Department of Cell Stress Biology Photodynamic Therapy Center Roswell Park Comprehensive Cancer Center Buffalo NY 14203 USA; ^5^ Department of Pharmacodynamics and Molecular Pharmacology Faculty of Pharmacy Collegium Medicum in Bydgoszcz Nicolaus Copernicus University in Torun Bydgoszcz 85‐067 Poland

**Keywords:** drug monitoring, in vivo lung perfusion, in vivo sampling, solid‐phase microextraction, tissue sampling

## Abstract

Chemical biopsy by solid‐phase microextraction (SPME) with sorbent‐coated fibers offers monitoring of biological processes in a significantly less invasive manner compared to conventional tissue biopsy. The developed device features a self‐protective design by using an acupuncture needle coated with biocompatible material only in a recessed section. The coating comprises micron‐sized naturally wettable sorbent particles embedded in a durable, biocompatible binder, ensuring broad analyte extraction coverage without the need for solvent activation. This design allows professionals in biochemical research and medical staff to use the device for in vivo monitoring of tissue concentrations of endogenous and/or exogenous substances without introducing activation solvent to the investigated system. The device is successfully used to sample anti‐cancer drugs in both animal models and human patients undergoing in vivo lung perfusion (IVLP) surgery, and then determine the drug concentration level by LC/MS. Finally, a proof‐of‐concept experiment using a microfluidic open interface (MOI) to directly desorb and introduce the extract analytes from the coating to MS detection is proposed for potential on‐site real‐time drug monitoring during the surgery.

## Graphical abstract

A new tissue sampling device using an acupuncture needle with bio‐compatible extraction sorbents in the recessed section is developed. The coating material using self‐wettable particles doesn't require solvent activation after sterilization. This device is applied for the anti‐cancer drug concentration monitoring during the in vivo lung perfusion surgery for clinical trial phase I studies.

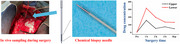

## 1 Introduction

Tissue biopsy is a widely used standard diagnostic procedure in various medical applications. However, its invasive nature can pose risks to patients due to potential organ damage, thereby restricting the scope, location, and timing of sampling. In vivo analysis via micro/nano analytical techniques enables accurate monitoring and prediction of processes occurring within complex living systems.^[^ ^1^, ^2^, ^3^ ^]^ Among current techniques, microdialysis (MD) sampling coupled with LC/GC‐MS analysis is favored for the determination of small molecules in tissue due to its ability to provide comprehensive chemical information.^[^ ^4^, ^5^ ^]^ Nevertheless, challenges persist in sampling nonpolar compounds, such as nonpolar drugs and lipids^[^ ^6^, ^7^, ^8^ ^]^ which are often highly bonded to the tissue matrix or adsorbed onto sampling devices. Additionally, further sample preparation might be required to enhance sensitivity and decrease matrix effects prior to instrumental analysis.^[^ ^9^, ^10^ ^]^ Low‐flow push‐pull perfusion offers an alternative method for in vivo sampling, providing better spatial and temporal resolution with minimal dilution ^[^ ^11^ ^]^ although it requires a more complex setup compared to MD.

Solid‐phase microextraction (SPME), wherein a small amount of biocompatible coating is immobilized onto a solid support to extract analytes from a given matrix, offers several advantages over traditional sampling methods, including simplicity, minimal invasiveness, and simultaneous clean‐up of extracts.^[^ ^12^, ^13^ ^]^ SPME has proven effective for simultaneous in‐vivo sampling and enrichment of fragrances and pheromone components emitted by plants and animals,^[^ ^14^, ^15^ ^]^ as well as compounds present in human breath and skin emissions.^[^ ^16^, ^17^ ^]^ By enriching and characterizing unstable compounds otherwise not easily captured by traditional methods, applications of SPME coupled with GC/MS have enhanced our current understanding of many biological processes and have resulted in the improvement of a variety of aroma products.^[^ ^18^ ^]^ Recent improvements in coating design have extended the application of SPME technology to in vivo biofluidic and tissue sampling.^[^ ^19^, ^20^ ^]^ The molecular filter function provided by polymeric binders, such as polyacrylonitrile (PAN),^[^ ^21^ ^]^ in biocompatible coatings, enables selective extraction of small molecules from a variety of complex sample matrices, including animal tissue. The small size of the coated wire format and the non‐exhaustive nature of SPME result in negligible perturbation to the living system. SPME extracts only a small fraction of compounds based on their availability and affinity toward the sorbent via the free concentrations within the investigated living system. Quantitative analysis can be done either in accordance with their diffusion coefficients for short sampling times, or distribution constants in multiphase systems once partitioning equilibrium is reached. Previous metabolomics investigations have shown that insertion of a small SPME device into tissue does not result in detectable stress or tissue damage during sampling, as no specific stress chemicals were released during needle insertion.^[^ ^22^ ^]^ However, despite over a decade of research, spanning from in vivo animal studies to human applications, several issues and challenges were raised by surgeons during the clinical trial studies, mainly about the currently available SPME device using nitinol fiber coated with sorbent materials.^[^ ^23^ ^]^ These include (1) convenience of use: in practice, there is no handle for the SPME fiber, and the nitinol fiber is too easy to bend. The fiber is dark in color, make it difficult to observe after insertion into tissue; (2) Using a sheath tubing or cannula is often required during puncturing to protect the coating material, complicating the process and increasing invasiveness; (3) Coating activation: while activating the coating with the use of organic solvents can enhance extraction efficiency and reproducibility, this method is not permissible for human applications post‐sterilization.

In this work, for solving the above challenges related to in vivo tissue sampling, the first tailored SPME device was developed. This device is based on a medical‐grade acupuncture needle with coating material only in a recessed section. During the puncturing, the needle tip can protect the coating material, without the need for sheath tubing. Besides, a self‐wettable coating material was developed by embedding wettable sorbent particles in the polymeric binder, which ensures the device can be directly deployed in vivo for human applications without solvent activation. This device is aimed as a single‐use medical device and has been successfully tested in both animal models and human clinical phase I trials for in vivo sampling at various time points and spatial locations during the surgical treatment. The anti‐cancer drug concentration levels in tissue were determined by further solvent desorption and LC‐MS analysis.

## 2 Results

### 2.1 Fabrication and Testing of Coated Acupuncture Needle

**Figure 1:**
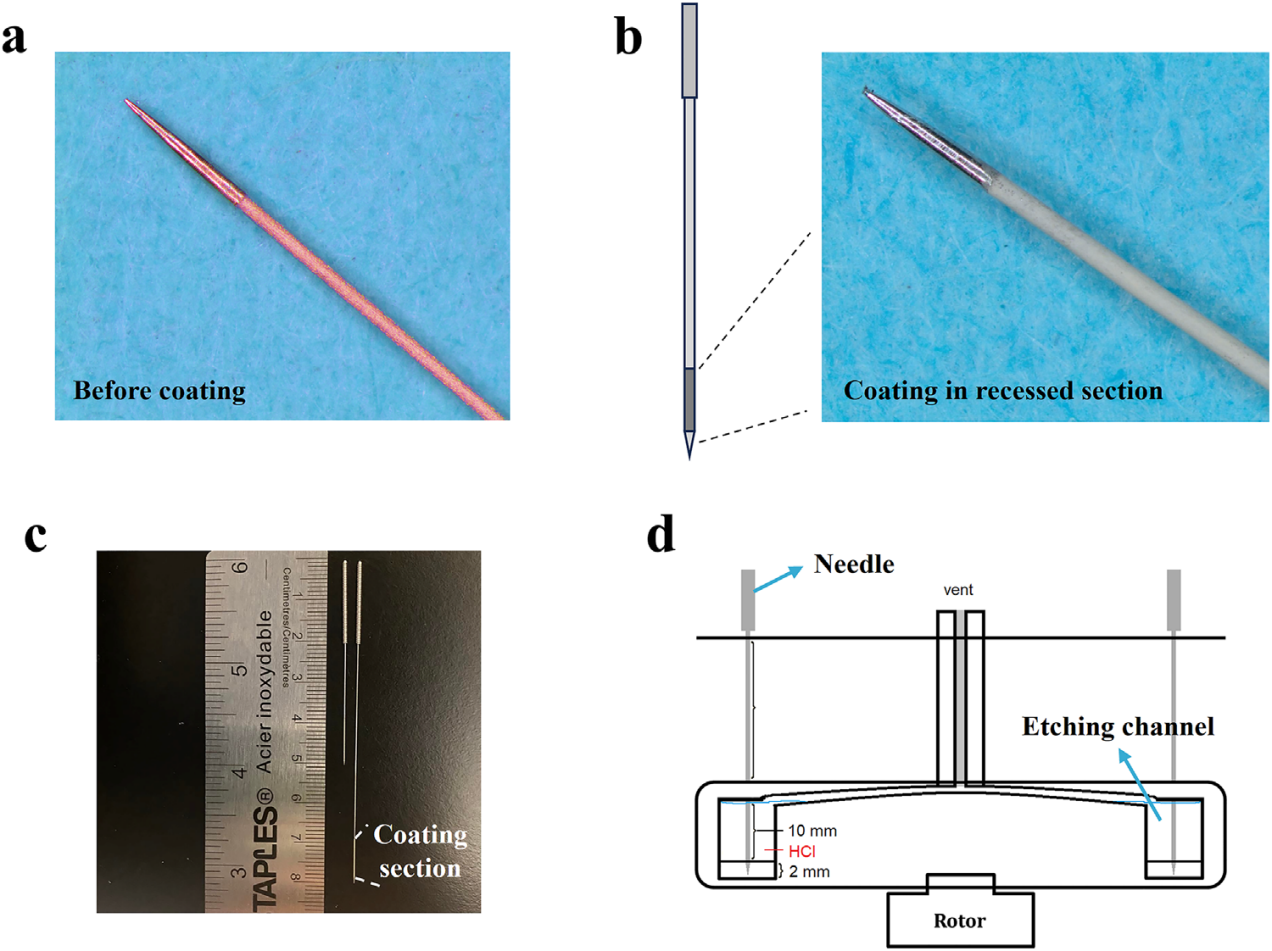
a) Acupuncture needle with recessed section before coating. b) Acupuncture needle with extraction coating in the recessed section. c) Photo of the recessed acupuncture needles with two different lengths. d) Schematic of the chemical etching station with the capacity of etching 20 needles simultaneously.

To achieve effective in vivo sampling of tissue, an SPME device requires great robustness in both its supporting fiber core and coating material to reliably puncture organ surfaces. In this work, a medical‐grade acupuncture needle made of surgical‐grade stainless steel with a diameter of 250 µm was selected as the supporting fiber cone. As depicted in **Figure** **1**a, a recessed section with a diameter of 220 µm was chemically etched using hydrochloric acid below the tip. The extraction material was coated exclusively within this recessed section, with a thickness of 15 µm using dip‐coating method (Figure 1b,c). More microscope images of the detailed structure were shown in Figure S1 (Supporting Information). In this design, during puncturing, the SPME coating is mechanically shielded by the uncoated tip, eliminating the need for previously used sheath tubing or guide cannula for protection during insertion and removal of the SPME fiber. The addition of a spring handle on the acupuncture needle facilitates convenient handling during sampling. These modifications serve to increase the robustness, simplify the procedure, and reduce invasiveness when compared with previous in vivo SPME devices. In addition, the acupuncture needle made by stainless steel is easy to observe by its color compared with the previously used nitinol wire.

The first challenge we faced was producing a highly precise and reproducible recessed section on commercially available acupuncture needles in a relatively high‐throughput manner. As shown in Figure 1d, S2a,b (Supporting Information), a vertical chemical etching station was developed that enables the simultaneous production of 20 acupuncture needles. During the chemical etching with HCl, the tip was protected by inserting it into the rubber placed at the bottom of the station. The etching channel was filled up using highly concentrated HCl. During the examination, the quality of the acupuncture needle is the most important factor for preparing a reproducible diameter of recessed section, please follow our suggestion in the experimental section if you want to use this design. Using a rotor, the device was rotated clockwise for 3 s and then counterclockwise for 3 s to accelerate the etching and improve the reproducibility. We tested 3 batches, 60 needles in total, measuring the recessed section diameter after etching (Table S1, Supporting Information). Using 220 µm as an internal reference diameter, needles deviating more than ±5 µm were classified as unqualified. Among the 60 needles tested, only 4 were unqualified, yielding an overall rejection rate of 6.7%. Including the unqualified needles, the intra‐batch relative standard deviations (RSD%) for diameter were ≤ 1.37%, and the inter‐batch RSD% was 1.46%. Excluding the unqualified needles, intra‐batch RSD% improved to ≤0.86%, and inter‐batch RSD% was 0.96%. The lengths of the recessed sections are adjustable by changing the length of the etching channel. Currently, two different recessed section lengths, 5 and 10 mm, are available with three different needle lengths, including 5, 8, and 17.5 cm for different applications (Figure S2c,d, Supporting Information).

**Figure 2:**
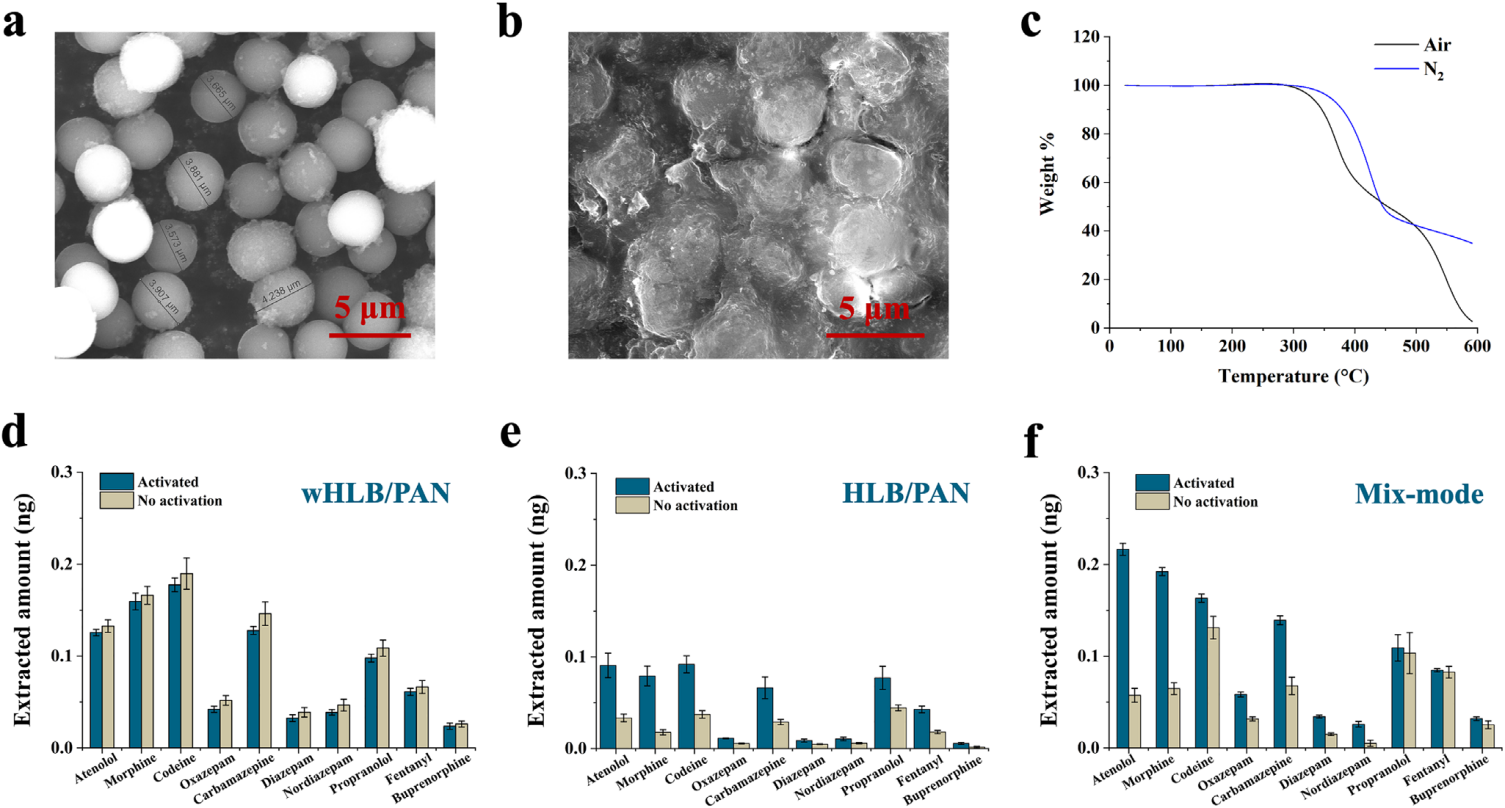
a) SEM image of the wHLB particles. b) SEM image of the wHLB/PAN coating. c) Thermogravimetric analysis of wHLB particles in air and nitrogen. d) extraction performance of the wHLB/PAN coating with and without activation by organic solvents. e) and f) Extraction performance of regular HLB/PAN coating e) and mix‐mode bio‐SPME fiber f) with and without activation by organic solvents. The peak areas of the testing drugs by LC‐MS were converted to exact amounts by instrumental calibration curves, explained in the experimental section in Supporting Information. Data points represent mean values ± standard deviation (SD) of three technical replicates.

Another major limitation of currently used SPME devices for in vivo analysis is the requirement for activating the sorbent coating material prior to sampling to ensure optimal extraction efficiency and reproducibility. This activation typically requires organic solvents, such as methanol or acetonitrile mixed with water (e.g., methanol/water 1/1, V/V), to interact with the coating material before sampling. For medical applications, no solvents are permitted to come into contact with medical devices post‐sterilization via autoclaving. However, the autoclaving at high temperature removes the solvents inside the coating, causing lower extraction efficiency and decreased reproducibility. A new type of sorbent particle with a self‐wettable feature, named wettable hydrophilic‐lipophilic balance material (wHLB), was developed by modifying the chemical synthesis conditions of regular HLB particles. By altering reaction conditions, including the monomer ratio (N‐vinylpyrrolidone vs divinylbenzene), reaction temperature, reaction solvents, and reaction vessel, uniformly sized wHLB particles with an average diameter of 4 µm were synthesized (**Figure** **2**a). The dynamic light scattering (DLS) data in water, shown in Figure S3a (Supporting Information) showed that the hydrodynamic diameter of the wHLB particles in water has an average size of 4.16 µm. SEM image of the wHLB/PAN coating material on the acupuncture needle (Figure 2b) suggested that when using PAN as the binder, the wHLB particles were embedded into the binder. More SEM images of the coated needle are shown in Figure S3b (Supporting Information). Figure 2c demonstrates good thermal stability up to 300 ˚C in both air and nitrogen gas conditions, which is stable in the autoclave. The contact angle testing was used to assess the wettability of the coating surface. As shown in the Figure S4 (Supporting Information), the regular HLB/PAN has a contact angle of 70.3˚, while the wHLB/PAN coating has the contact angle of 26.0˚, demonstrating the wHLB/PAN has a more hydrophilic surface compared with regular HLB/PAN. Elemental analysis (Table S2, Supporting Information) revealed an increased nitrogen ratio corresponding to the amount of N‐vinylpyrrolidone used in the synthesis, which is responsible for the wettable feature of wHLB.

The bio‐compatible coating was prepared by mixing sorbent particles with the polyacrylonitrile (PAN) to form a homogeneous slurry and then coating the needle by the dip coating method. The slurry is stable in room conditions at least half a year under stirring and can be used repeatably. The PAN here works not only as glue, but also as a molecule filter that only allows small molecules to enter the coating and encounter the wHLB particles. This avoids the binding and interference from larger molecules present in the tissue matrix, as being demonstrated in our previous works.^[^ ^21^, ^24^, ^25^ ^]^ The interactions between different sorbent particles and binders have been studied in our previous work and won't be the focus of this study.^[^ ^26^ ^]^ After manufacturing the coated acupuncture needles, the mechanical robustness of the coated needles was tested by directly puncturing through the PTFE septa (3.5 mm in thickness) used in GC inlet, pig heart, and liver. No visible damage to the coating was observed under a microscope (Figure S5, Supporting Information). The extraction efficiency of the coated needle before and after puncturing was assessed by analyzing 10 different drugs spiked in PBS solution, revealing no change in the mean amount extracted (Figure S6, Supporting Information). The extraction performance of the SPME device, with and without solvent activation, was evaluated. As shown in Figure 2d, the wHLB/PAN coating demonstrated good reproducibility with RSD% ≤ 14% (n = 4) for all 10 compounds in lamb lung tissue sampling, with fewer differences observed in extracted amounts between activated and non‐activated SPME needles. The regular HLB/PAN and the commercial mix‐mode bio‐SPME coatings were also tested with and without activation and the results are shown in Figure 2e,f, which clearly demonstrates that without solvent activation, these two coatings performed much worse extraction efficiency and higher RSD% when compared with after solvent activation. The mix‐mode/PAN bio‐compatible material is a coating material for the commercial SPME fiber and was used in most of the previous in vivo SPME applications. The extraction of this sorbent is based on both hydrophobic and cationic exchange interactions. For this reason, it performed better extraction efficiency for polar and positively charged drugs such as atenolol and morphine after solvent activation. However, without activation, wHLB/PAN performs better extraction efficiency for most of the drugs because of the self‐wettable feature. The extraction performance of wHLB/PAN coating in other biological samples such as human whole blood, was also tested and shown in Figure S7 (Supporting Information), good reproducibility and extraction efficiency can also be obtained without solvent activation, suggesting the potential of using this device for the on‐site sampling of other bio‐samples. The extraction coverage and efficiency of the wHLB coating were further compared with commercial C18, mixed‐mode, and HLB coatings (Figure S8, Supporting Information) using a group of 28 drugs with log P values ranging from 0 to 6 in human plasma samples. The results indicated that the wHLB/PAN coating provides broad extraction coverage with superior extraction efficiency for most analytes, outperforming commercially available coating materials.

### 2.2 In Vivo Tissue Analysis Using Coated Acupuncture Needle

**Figure 3:**
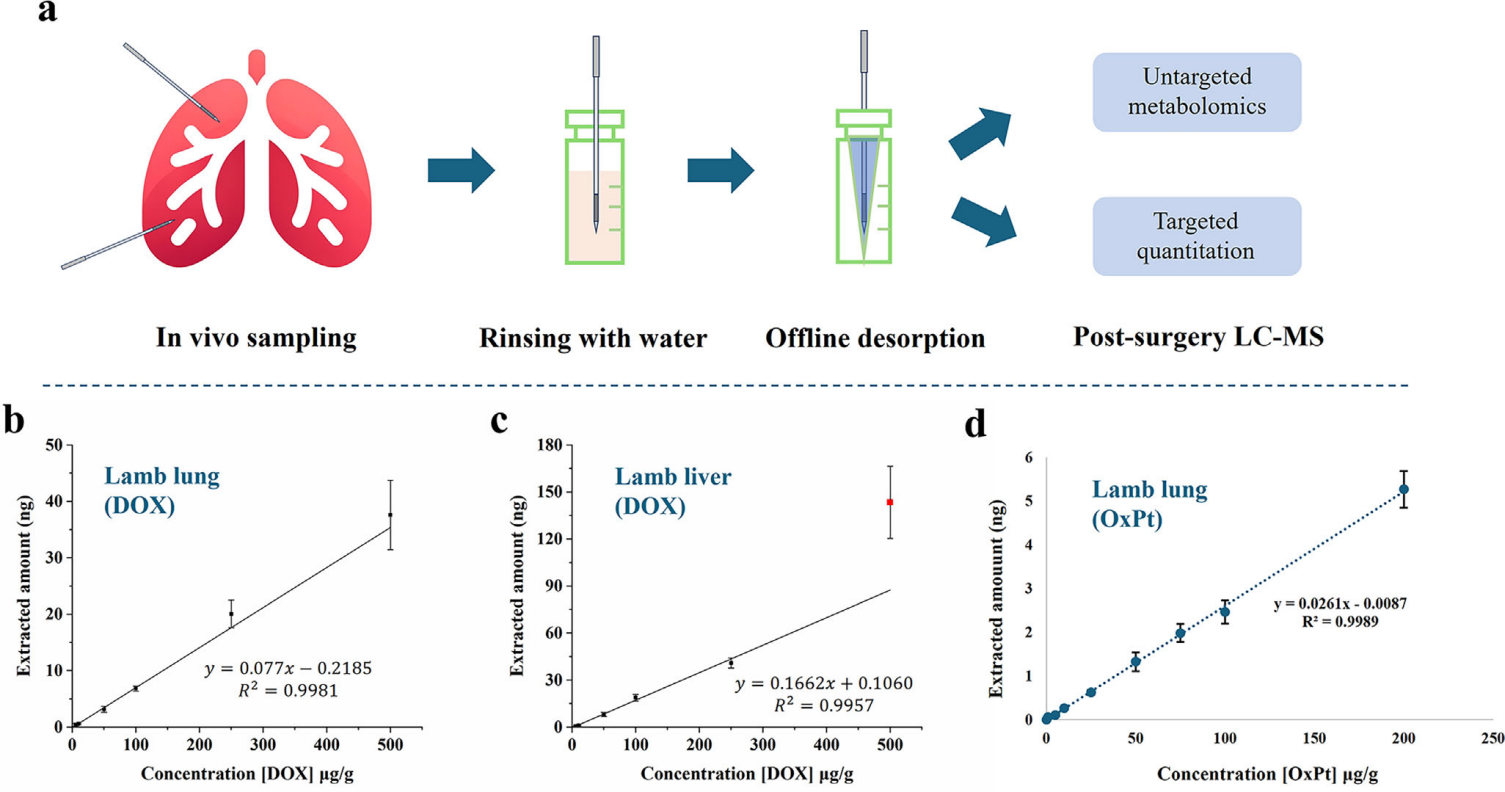
a) Schematic of in vivo sampling coupled with post‐surgery LC‐MS analysis for untargeted metabolomics and targeted quantitative analysis. b‐d) Ex vivo calibration curves of DOX in lamb lung b), lamb liver c), and OxPt in lamb lung d) using LC‐MS. Data points represent mean values ± standard deviation (SD) of four technical replicates.

By coupling in vivo SPME sampling with MS‐related detection techniques such as LC‐MS, both targeted quantitation and untargeted qualitative analysis are enabled (**Figure** **3**). In our previous works, we have demonstrated that combining in vivo SPME with untargeted LC‐MS, we are able to monitor the metabolomics’ changes during the in vivo lung perfusion (IVLP) lung cancer surgery process or during the organ transplantations.^[^ ^27^, ^28^ ^]^ In this work, we are focusing on demonstrating this new device in the target quantitative analysis of chemotherapy drugs in vivo.

As being demonstrated from our previous work using pig mode, ex vivo matrix‐match calibration using spiked surrogate matrices can be employed to quantify two anti‐cancer drugs, doxorubicin (DOX) and oxaliplatin (OxPt), for in vivo tissue analysis.^[^ ^29^, ^30^, ^31^ ^]^ For matrix‐match calibration, to ensure the similar binding effects of targeted analytes with matrices, different surrogate matrices from lamb need to be used for different parts of human organs, and spiked with targeted analytes at different concentration levels to make the calibration curve. As the clinical human samples usually come at different dates, to avoid the variation of sensitivity from LC‐MS, the exact amount (ng) of the analytes in the desorption solution was calculated by external calibration of the instrument instead of using peak area. Lamb lung was selected as the surrogate matrix for the quantitation of target drugs for an in vivo human lung study. Ex vivo calibration demonstrated linearity from 10 to 500 µg g^−1^ with a linear correlation coefficient (R^2^) of 0.9981 and a limit of detection (LOD) of 5 µg g^−1^, and a limit of quantitation (LOQ) of 10 µg g^−1^ (Figure 3b). Lamb liver was used as the surrogate matrix for DOX analysis in liver tissue (Figure 3c). Unlike in the lung, DOX spiked in liver tissue lost linearity at a concentration of 500 µg g^−1^, suggesting saturation and disruption of the adsorption equilibrium between DOX and the liver tissue. As in the chemotherapy, the drug concentration in tissue is much lower than this level, so it won't be an issue for in vivo quantitation. The method was also utilized to create a matrix‐matched calibration curve for OxPt in lamb lung tissue, and the data is shown in Figure 3d. The LOQ is 1 µg g^−1^ for OxPt. It should be mentioned that OxPt is not stable when stored in room temperature, as we tested, analysing the sample using LC‐MS within 12 h after defrosting is necessary.

**Figure 4:**
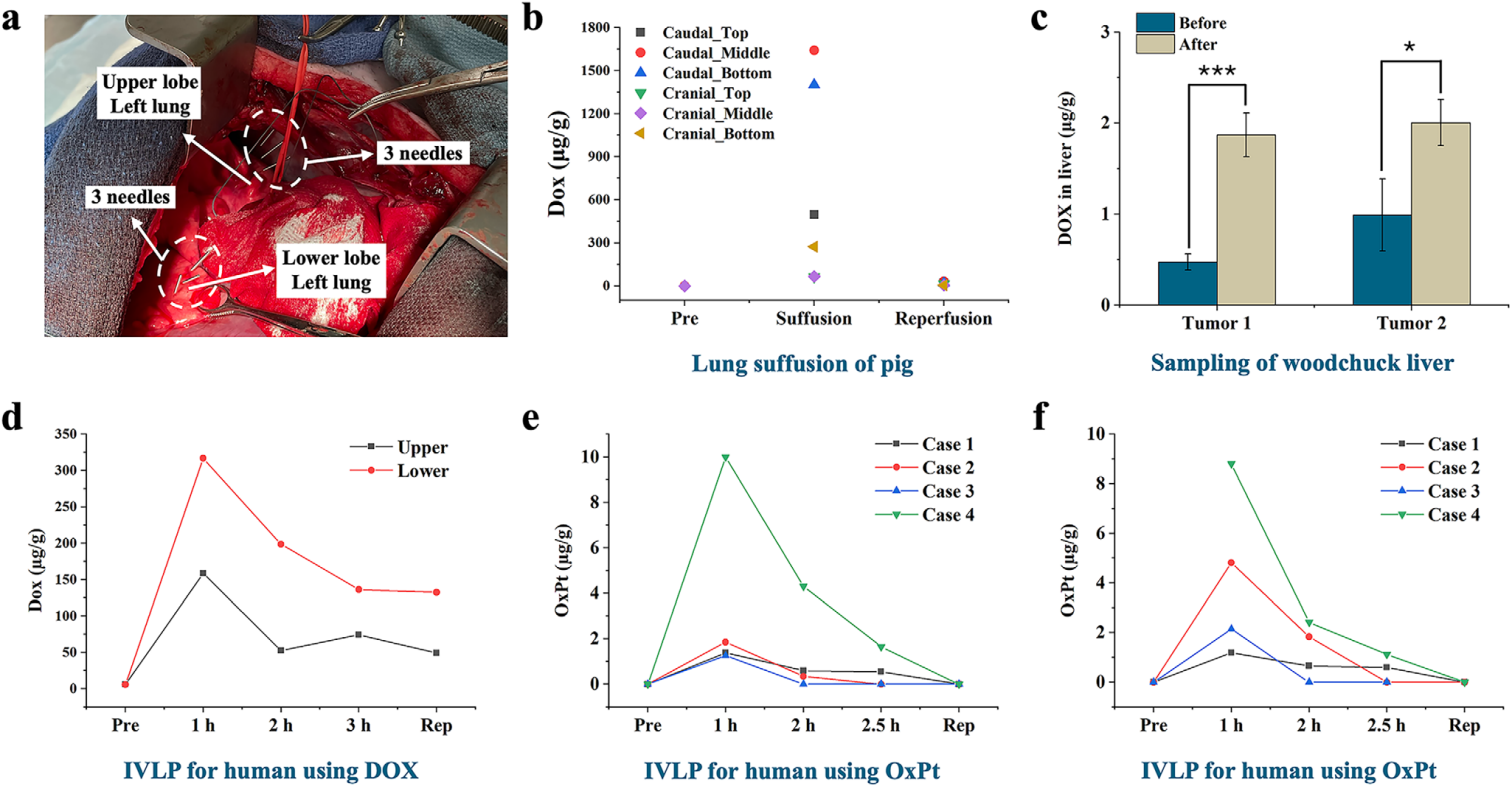
a) Photo of in vivo sampling in the pig left lung during IVLP treatment with DOX. Three needles were inserted in the upper lobe, and three needles were inserted in the lower lobe. b) DOX concentration levels during localized chemo‐suffusion treatment of DOX in a pig. c) DOX concentration levels during localized interstitial chemo‐phototherapy (I‐CPT) with PhotoDOX in woodchuck liver tumors. Data points represent mean values ± standard deviation (SD) of three in vivo sampling replicates in the same tumor area. *p < 0.05, **p < 0.01, ***p < 0.001. d) DOX concentration levels in human left lung tissue during IVLP treatment of lung sarcoma metastases. e, f) OxPt concentration levels in human left lung tissue during IVLP treatment of colorectal metastases, e) upper lobe and f) lower lobe.

In the first application, the SPME needles were used for in vivo quantitative analysis of DOX concentrations in tissue for a preclinical study conducted in the lung of a pig (**Figure** **4**a). The pig underwent localized chemo‐suffusion with DOX via pulmonary bypass with shunts and balloons for in vivo chemo‐suffusion surgery.^[^ ^32^ ^]^ No acute trauma, bleeding, or short‐term adverse effects were observed during in vivo SPME sampling. Lung tissue DOX concentrations were determined via matrix‐matched calibration and are shown in Figure 4b. As expected, no DOX was detected before the suffusion. After suffusion for 10 min, significant tissue concentrations of DOX were detected. An uneven distribution of DOX in the lung tissue was observed, likely due to the static suffusion method used for drug delivery in this study. This large spatial variability was expected given the passive nature of suffusion compared to active chemoperfusion approaches such as in vivo lung perfusion (IVLP),^[^ ^30^ ^]^ which promotes evenly distributed delivery of the drug in the lung. However, lung suffusion is less invasive for patients compared to IVLP. Lastly, low levels of DOX were also detected following the washing out step during blood reperfusion.

In the second application, the SPME needles were also employed to investigate the effects of localized interstitial chemo‐phototherapy (I‐CPT) with PhotoDOX in woodchuck liver tumors. PhotoDOX is a phospholipid nanoparticle that incorporates a photosensitive drug in its phospholipid bilayer, loaded with DOX.^[^ ^33^ ^]^ Upon light activation of PhotoDOX, DOX is released within the target tumor volume. SPME probes were inserted into two liver tumors in woodchucks before and after IPDT light activation. Tissue DOX concentrations were determined via matrix‐matched calibration and are shown in Figure 3c. The results demonstrated that local tissue DOX levels increased significantly following localized light activation of PhotoDOX.

Building on pre‐clinical studies and our prior research using commercial bio‐SPME fiber for human studies, the coated acupuncture needles were deployed during surgical procedures in a phase I clinical trial of DOX delivery via in vivo lung perfusion (IVLP) for treatment of lung sarcoma metastases.^[^ ^34^ ^]^ Sterilized needles were individually inserted into the upper and lower lobes of the left lung at 5 sampling time points. No bleeding or adverse effects were observed at the sampling sites during surgery, and there were no intraoperative complications. As depicted in Figure 4d, tissue concentrations reached 351.1 µg g in the lower lobe and 148.4 µg/g in the upper lobe after 1 h of IVLP, and then gradually decreased over the perfusion period. Following a 30‐min post‐blood reperfusion, concentrations decreased to 128.8 µg g^−1^ in the lower lobe and 47.7 µg g^−1^ in the upper lobe. The observed residues in the lung after reperfusion are likely due to the high matrix binding of DOX to the tissue. Results also indicated varying concentration levels of DOX in different sections of the lung.

The in vivo sampling method was also applied in another human study that included four cases undergoing a phase I clinical trial using OxPt for IVLP treatment of colorectal metastases in the lung. Concentration levels in the upper and lower lobes of patients’ lungs at different time points are shown in Figure 3e,f. Similar to DOX during IVLP, the highest concentration level was observed at 1 h for all cases. In comparison to DOX, the tissue concentration level of OxPt was much lower than DOX after 1 h of IVLP, due to the fast pharmacokinetic of OxPt in lung tissue compared to DOX. These have been studied and reported in the previous papers.^[^ ^35^, ^36^ ^]^ Data from previous and the currently described study suggested the tissue concentration levels of OxPt were low after 2 h perfusion, providing valuable information to the medical team, helping them decide to decrease perfusion time from the initial 3 to 2.5 h during the clinical trial.^[^ ^35^ ^]^ The patients’ information related to the human clinical trial studies are listed in Table S2 (Supporting Information). It is worth mentioning that pneumothorax, a significant clinical risk factor following percutaneous lung biopsy, occurs in about 27.3% of cases, with 25.1% of incidents happening during follow‐ups.^[^ ^37^ ^]^ Due to the low invasiveness of the in vivo SPME (both the previous bio‐SPME device and the current device), no cases of pneumothorax have been reported in the 25 human cases we have studied.

### 2.3 Potential Application in Real‐time Drug Monitoring During the Surgery

**Figure 5:**
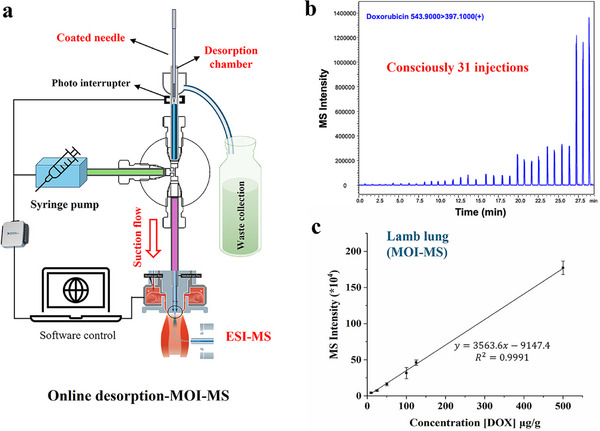
a) Schematic of the automatic MOI‐MS design. The system is controlled by custom software developed in Visual Studio. Online desorption of 10 s and MS detection were used for fast analysis. b) MOI‐MS spectrum of DOX during continuous injection of 31 samples over 30 min. c) Ex vivo matrix‐match calibration curve for OxPt using lamb lung as surrogate matrix, and then MOI‐MS analysis. Data points represent mean values ± standard deviation (SD) of four tissue sampling replicates.

Coupling in vivo SPME with direct MS analysis, without the need for LC separation, enables rapid and on‐site analysis. As shown in **Figures** **5**a and S9 (Supporting Information), a microfluidic open interface (MOI), which was developed from our previous work, enables the direct desorption of analytes from the coating into the open micro‐chamber with the use of 5 µL of desorption/ionization solution.^[^ ^38^ ^]^ The low volume of the desorption solution allows for rapid desorption equilibria within 10 s and a total analysis time of 1 min per sample.^[^ ^39^ ^]^ The MOI‐MS analysis workflow has been automated using a IR photo interrupt to record the flow in the chamber and a programmable syringe pump to control the flow rate in the system. Using the current design, the nurse only needs to put the needle into the chamber for 10 s and take it out, the MOI‐MS can automatically record the compounds signal and transfer to the concentration level in the tissue. As demonstrated in Figure 5b, the MOI‐MS system allows to continuous injection and analysis of the tissue concentration level of DOX after in vivo SPME sampling, with an average sample analysis time of 1 min per sample. The ex vivo calibration of DOX in lamb lung using MOI‐MS demonstrated excellent quantitation performance with an LOD of 3 µg g^−1^ (Figure 5c), comparable to results obtained via in vivo SPME‐LC‐MS. It should be clarified that this part of the study is just a preliminary concept and has been tested ex vivo, without clinical validation. Currently, our focus is on obtaining ethical permission for incorporating MS detection near the surgery operation room and automating the needle‐handling process after sampling. Various factors, including the interference from the surgical environments, ethical barriers to intraoperative MS use, need to be consulted and studied with the medical team before the clinical testing.

## 3 Discussion and Conclusion

In vivo SPME has been employed in various animal studies, including sampling chemically unstable oxylipins from the brains of awake, moving rats,^[^ ^7^ ^]^ analyzing pharmacokinetics in the veins of dogs,^[^ ^40^ ^]^ and examining neurotransmitters in the brains of awake monkeys.^[^ ^20^ ^]^ These animal studies have underscored the excellent biocompatibility of the method resulting in reduction of animal suffering and minimizing the use of life animals, while providing insight into true composition of small molecules in living systems. Following over a decade of animal research, recently, in vivo SPME has been applied in human clinical trials to monitor changes in exogenous drug concentrations and endogenous metabolites during clinical treatments for lung cancer and brain tumors.^[^ ^30^, ^41^ ^]^ However, significant challenges have emerged with currently available SPME devices, which are not optimized for in vivo human studies, particularly during surgical procedures, and have limited the applications of in vivo SPME. The main innovation of this work is developing the first tailored SPME device for in vivo tissue sampling, focusing on addressing the challenges raised by medical stuff during the human clinical research. Various new developments, including encompassing the design of the structure of the device to make it easier of handle, self‐protected, and synthesis the self‐wettable sorbent material have been done in this work. Altogether these improvements, this new single‐use in vivo SPME device received good comments from the surgeons who used them during the human clinical trials. In this study, only a few clinical cases were examined, only to demonstrate the analytical performance of this device. Extensive medical studies are not included in this work. In the future, more comprehensive clinical testing targeting specific diseases and targeted drugs should be done before it can be applied in different clinical analysis.

In vivo lung perfusion (IVLP) with DOX or OxPt is a novel approach for the treatment of lung metastases, enabling localized delivery of anticancer drugs to the lungs while minimizing systemic exposure and reducing toxicity to other organs. Our in vivo SPME sampling, coupled to LC‐MS analysis, can provide information of tissue concentration levels at several different time points during the surgery with minimal invasiveness to the patient. This information is essential for the medical doctor to study the correlations between dosage/tissue concentration level/perfusion time and patients’ outcomes, this has been studied and discussed by medical teams in the previous works,^[^ ^35^, ^36^ ^]^ and is not included in this paper.

The coupling of in vivo SPME with LC‐MS provides a robust method for studying general metabolic trends and investigating potential biomarkers but it lacks real‐time capabilities for point‐of‐care analysis. This limitation becomes important in the treatment of advanced‐stage cancer patients, where metabolic processes can vary significantly due to individual physiological conditions. For example, our pharmacokinetic data for DOX and OxPt revealed substantial variability influenced by patient‐specific factors, such as physiological state and cancer stage, among others. Coupling in vivo SPME with direct/ambient MS techniques, such as MOI‐MS or portable MS offers promising solutions during surgery.^[^ ^42^ ^]^ Functionally, in vivo SPME‐direct MS can be a complemental technology with the intelligent knife and the mass spectrometer pen,^[^ ^43^, ^44^ ^]^ with lower time and spatial resolution, but offers deeper organ insights beyond surface analysis as well as quantitative features using different calibration methods. Its selective extraction and clean‐up capabilities result in cleaner extracts with low matrix effects with contamination to the MS instrument. However, for on‐site application in the surgical room, various factors, including the interference from the surgical environment, ethical barriers to intraoperative MS detection, need to be consulted and studied with the medical team before further clinical testing.

The application of in vivo SPME chemical biopsy tools in clinical analysis and diagnosis is currently facilitated by locally issued ethics permits. However, the number of hospitals/patients involved in such research remains limited due to the lack of standardized devices legally endorsed for use as medical tools. We believe the tailored device developed in this work has the potential to overcome this limitation, promoting the adoption of in vivo SPME chemical biopsy in clinical practice. This medical application is driven by the integration of this chemical biopsy tool with analytical instrumentation for quantitative readout. Advanced analytical detection instruments, such as portable MS, high‐resolution MS, ion mobility‐MS, ambient/direct MS, as well as Raman, fluorescence, and electrochemical sensors,^[^ ^45^, ^46^, ^47^, ^48^ ^]^ can further improve the feasibility of in vivo SPME method in clinical applications, facilitating personalized medicine.

## 4 Experimental Section

### General Devices and Chemicals

Seirin Laser SL2530 Stainless steel acupuncture needles, 30 mm × 0.25 mm and 60 mm × 0.25 mm, were purchased from Eastern Currents (Vancouver, BC, Canada). Carbo CT1 stainless steel acupuncture needles, 125 mm × 0.315 mm, were purchased from Opis Supplies (Markham, ON, Canada). The quality of stainless‐steel needles was one of the most important factors for the reproducibility of the etching process. Drug standards, including caffeine, cotinine, atenolol, acetaminophen, nicotine, codeine, heroin, amphetamine, metoprolol, methamphetamine, MDMA, oxazepam, cocaine, lorazepam, tramadol, clonazepam, carbamazepine, diazepam, nordiazepam, LSD, venlafaxine, propranolol, citalopram, methadone, paroxetine, fentanyl, and buprenorphine, were purchased from Cerilliant Corporation (Round Rock, TX, USA). Acetonitrile (HPLC grade for particle synthesis), toluene (HPLC grade for particle synthesis), divinylbenzene (DVB), N‐vinylpyrrolidone (NVP), 2,2'‐azobis(2‐methylpropionitrile) (AIBN), polyacrylonitrile (PAN), hydrochloric acid (HCl), dimethylformamide (DMF), and phosphate buffered saline were purchased from Millipore Sigma (Burlington, MA, USA). Formic acid (FA), sodium hydroxide, and LC‐MS grade mobile phases including methanol (MeOH), ACN, 2‐propanol (IPA), and water were purchased from Fischer Scientific (Mississauga, ON, Canada). C8‐SCX mixed‐mode fibers and particles were kindly provided by Supelco (Milwaukee, PA, USA). Doxorubicin (DOX) and oxaliplatin (OxPt) were purchased from Toronto Research Chemicals (Toronto, ON, Canada). Lamb lungs and livers were purchased from a local butcher shop (Waterloo, ON, Canada). The Kinetex PFP column (2.1 mm × 100 mm) with a 1.7 µm particle size was purchased from Phenomenex (Torrance, CA, USA).

### Fabrication and Testing of Devices—Etching of Acupuncture Needles

A vertical etching station (Figure S2a,b, Supporting Information) featuring a rotor that alternates the rotation of the etching chamber in clockwise and counterclockwise directions was constructed. The space between the top and bottom pieces of the etching station was 5 or 10 mm, depending on the length of the recessed section. The tip was protected by the acid‐resistant rubber mat. The rotor was programmed to spin 1.5 revolutions every 3 s. A 15 µm recession can be obtained using 100 mL of HCl (37% w/w) for 30 min. After removing the HCl, the recessed needles were rinsed with water, submerged in MeOH, sonicated in a water bath for 15 min, and dried by N_2_ prior to coating.

### Fabrication and Testing of Devices—Synthesis and Characterization of wHLB Particles

Wettable‐HLB (wHLB) particles were synthesized via precipitation polymerization, developed based on our previous study,^[^ ^49^ ^]^ with further optimization to provide the wettability feature of the coating material. In a 200‐mL Teflon (PTFE) cylindrical reaction vessel (inner diameter 4.5 cm), 80.0 mL of ethanol was added as solvent, and 20.0 mL of toluene served as porogen. The solvent‐porogen mixture was continuously stirred with a 3 cm magnetic stir bar at 150 rpm and purged with N_2_ for 60 min while maintaining the reaction temperature at 80 °C in a silicon oil bath. Once the temperature of the oil bath stabilized, 2 mL of DVB, 6 mL of NVP hydrophilic functional monomer (purified by passing through Al_2_O_3_ to remove stabilizers), and 20 mg of AIBN initiator were added to the mixture to initiate the reaction. The reaction mixture was maintained at 80 °C and stirred at 150 rpm for 23 h. After this period, the reaction was quenched by placing the reaction vessel in a cold bath. The reaction mixture was then aliquoted into 50 mL Falcon centrifuge tubes and subjected to centrifugation for 20 min at 7000 rpm to collect the particles. The particles were washed 3 times with EtOH and subsequently collected via suction filtration (0.45 µm pore size). Lastly, the wHLB particles were dried at 60 °C under N_2_‐purged vacuum overnight.

For characterization, scanning electron microscopy (SEM) imaging was performed using a FEI Quanta Feg 250 ESEM (Hillsboro, OR, USA). Elemental analysis for carbon and nitrogen was conducted using a Costech 4010 Elemental Analyzer (Dar es Salaam, Tanzania) coupled to an Isochrom continuous flow isotope ratio mass spectrometer (Micromass, UK), with 5–10 mg of particles used for each analysis. Dynamic light scattering (DLS) measurements to determine the particle size of wHLB particles suspended in water were conducted using a Malvern Zetasizer Nano series analyzer (Malvern, UK). Thermogravimetric analysis (TGA) was performed on a TA Q500 instrument, with the temperature ramped at 20 °C min^−1^ from room temperature to 600 °C under N_2_ and air, respectively. For testing the contact angle of the coating materials, the coatings were prepared on the surface of a stainless‐steel blade, with a coating thickness of 10 µm. The contact angle was tested by RAMEHART‐contact angle measuring machine (Succasunna, NJ, USA) using standard protocol.

### Fabrication and Testing of Devices—Coating the Acupuncture Needles

The chemical sorbent was deposited into the recessed section of the needles via dip‐coating.^[^ ^50^ ^]^ A 7% (w/w) polyacrylonitrile (PAN) binder was prepared by dissolving 5 g of PAN in 70 g of DMF by heating at 85 °C and periodically stirring for ≈1 h until PAN was completely dissolved. Next, 0.65 g of the wHLB particles were added to 5.85 g of the PAN binder (10% w/w) in a 10 mL glass vial. The vial was sealed, and the mixture was submitted to mixing with a stir bar until use. A Nadetech NDDC CABIN dip coater was employed to quickly lower the needles into the slurry at a rate of 200 mm min^−1^ to ensure the recessed section was fully covered. After submersion, the dip coater held the needles in the slurry for 2 s before withdrawing them slowly at 90 mm min^−1^. The coated needles were then cured at 80 °C in an oven for 30 s. This process was repeated three times to achieve a coating thickness just below the depth of the recess. Finally, any coating left on the needle tips was removed by fine‐grit sanding. A 70% ethanol solution was employed to clean the coatings. Subsequently, the fibers underwent a standard medical sterilization procedure by steam in an autoclave at 121 °C for 45 min. The autoclaved SPME needles were then packed in sterile packaging and stored until use.

### Fabrication and testing of devices—Comparing Different Coating Materials with and without Activation

Three extraction phase sorbents—wHLB, HLB, and mixed mode (MM) particles—were directly compared to assess their extraction efficiencies with and without solvent activation. Four probe replicates of each sorbent were coated to a length of 1 cm and a thickness of 15 µm. Each probe was used to extract from 1.5 mL of sample matrix containing 50 ppb of drugs in homogeneous lamb lung tissue. The extraction was conducted for 20 min at 20 °C. Post‐extraction, each probe was rinsed with water for 5 s using a vortex mixer at 1500 rpm. The probe was then desorbed in 100 µL of a desorption solution (4/3/3 v/v MeOH/ACN/H_2_O) for 30 min at 500 rpm. Finally, 10 µL of each desorbed sample was analyzed via LC‐MS/MS. To evaluate the extraction coverage of the wHLB/PAN coating, 27 drug standards were spiked into human plasma at a concentration of 10 ng mL^−1^. The extraction time was 30 min with a vortex speed of 1000 rpm. After a brief 5 s wash with water, the needles were desorbed in 200 µL of MeOH/ACN/H_2_O 7/2/1 (v/v/v) for 30 min at 500 rpm. The desorption solution was then analyzed via LC‐MS/MS.

### LC‐MS and MOI‐MS Protocols

LC‐MS and MOI‐MS Protocols—LC‐MS/MS Analysis

A Thermo Vanquish UHPLC coupled to a Thermo TSQ Quantiva triple quadrupole mass spectrometer using heated electrospray ionization (HESI) (Thermo Scientific, San Jose, CA, USA) was employed for this study. For the MS, the ion transfer tube and vaporizer temperature were set at 325 and 350 °C, respectively. Sheath gas, auxiliary gas, and sweep gas flow rates were set at 50, 10, and 1 arbitrary unit (a.u.), respectively. The ESI voltage was maintained at +3.5 kV. A dwell time of 5 ms was set for each multiple reaction monitoring (MRM) transition. MRM information for the drugs was provided in a previous study.^[^ ^51^ ^]^ For the analysis of 10 drugs used in the coating comparison study, a Phenomenex PFP (2.1 mm x 10 mm, 1.7 µm) column was employed. Mobile phase A consisted of water with 0.1% FA, and mobile phase B was ACN with 0.1% FA. The flow rate was kept at 0.3 mL min^−1^. After 1 min of 10% B for column equilibration, the gradient was increased linearly to 95% B over 7 min, was held at 95% B for 2 min, and then dropped back to 10% B over 0.3 min, where it was maintained for 2 min. For the analysis of 27 drugs, a Waters C18 column (BEH C18, 10 mm length × 2.1 mm diameter, 1.7 µm particle size) was used. The mobile phase A was a mixture of 99% H_2_O +1% MeOH, containing 2 mm NH_4_AC, and B was a mixture of 90% MeOH+10% H_2_O, containing 2 mm NH_4_AC at a flow rate of 0.25 mL min^−1^. The gradient program was as follows: 0‐0.7 min, 30% B; 0.7–4.0 min, 30‐98% B; 4.0–12.0 min, 98% B; 12.0‐12.5 min, 98‐30% B; 12.5‐17.0 min, 30% B. For DOX analysis, the Phenomenex PFP (2.1 mm x 10 mm, 1.7 µm) column was used. The mobile phase A was H_2_O with 0.1% FA, whereas mobile phase B was ACN with 0.1% FA. The flow rate was 0.4 mL min^−1^. The gradient program was as follows: 0–0.5 min, 10% B; 0.5–4.5 min, 10‐95% B; 4.5–6.8 min, 95% B; 6.8–6.85 min, 95%–10%; 6.85–8 min, 10% B. For OxPt analysis, a Xbridge HILIC column (2.1×100 mm, 3.5 µm; Waters Corporation, Milford, MA, United States) was used.^[^ ^31^ ^]^ Mobile phase A was 50/50 v/v IPA/H_2_O with 5 mm NH_4_AC, and mobile phase B was ACN. The flow rate was 0.4 mL min^−1^. The gradient program was set as follows: 0–1 min, 95% B; 1–1.3 min, 95%‐40% B; 1.3–3.5 min, 40% B; 3.5–4 min, 40‐95% B; 4–6.5 min, 95% B. The injection volume for all described samples was 10 µL.

### LC‐MS and MOI‐MS Protocols—SPME‐MOI‐MS/MS for Fast Analysis

Detail of the MOI‐MS setup was outlined in a previous publication.^[^ ^39^ ^]^ A Shimadzu MS 8060 (Kyoto, Japan) triple quadrupole mass spectrometer was used for this experiment. A multi‐sample injection strategy was applied, allowing the MS instrument to continuously record signals while multiple samples were introduced in a single MS run. Following tissue extraction using coated acupuncture needles and a subsequent water wash, desorption was performed inside the MOI interface using a solution of MeOH/ACN/H_2_O 7/2/1 (v/v/v) with 0.1% FA. The MS continuously recorded during multiple needle desorption and injection. Each needle was introduced into the desorption chamber for 10 s before initiating the MOI software. Suction flow facilitated the transfer of desorbed analytes into the MS for signal generation. The MOI‐MS system features self‐cleaning and refilling functions, allowing for successive desorption and rapid analyses.

### Statistical Analysis

All data were presented as mean ± standard deviation (SD) and analyzed by Origin 2018 (software) to prepare the figures. The sample size (n) for each data point were clarified in the related figure captions. Student's t‐test was used to compare two groups. A p‐value less than 0.05 was considered statistically significant.

### Animal and Human Studies

### Animal and Human Studies—Ex Vivo Matrix‐Matched Calibration

For ex vivo matrix‐matched calibration in different tissue matrices, lamb lung and liver were used as surrogate tissues. Fresh whole tissue samples were homogenized using a blender with small amounts of dry ice to prevent heating of the tissue sample. Tissue homogenates were prepared and partitioned in a ratio of 1.5 mL of PBS (pH 7.4) to 13.5 g of blended tissue. The target analytes, DOX or OxPt, were spiked into 20 mL glass vials containing the tissue homogenate, followed by 1 min of manual mixing and 1 min of mixing on a benchtop vortex mixer at maximum speed. The final tissue concentrations for DOX were 0, 10, 25, 50, 100, and 500 µg g^−1^ in lung and liver homogenates. For OxPt, concentrations were 0, 5, 10, 50, 100, and 200 µg g^−1^ in lung homogenate. After getting equilibrium in the tissue, the spiked tissue homogenate samples were kept in a 37°C water bath during extraction. The extraction parameters were as follows: 20 min in static mode extraction, followed by a 10 s water wash with vortex agitation post‐extraction to remove any unspecific attachment and salts. Desorption was performed for 30 min with agitation at 500 rpm with MeOH/ACN/H_2_O 7:2:1 (v/v/v) with 0.1% formic acid solution for DOX and ACN/H_2_O 8:2 (v/v) solution for OxPt, followed with LC‐MS analysis. In the case of MOI‐MS, a 10 s static online desorption in the desorption chamber was used. As the clinical human case samples came from different dates over two years, the sensitivity of the LC‐MS system can vary a lot. The peak area of the matrix‐matched calibration curves data and clinical cases data were converted to the exact amount of the analytes in the desorption solution using an instrumental calibration curve (desorption solution spiked with drug standards at different concentration levels) during the injection sequence.

### Animal and Human Studies—In Vivo Sampling During Chemo‐Suffusion of Pig Lung

Following routine induction and endotracheal intubation for inhalation anesthesia with isoflurane, a 50 kg female purpose‐bred pig underwent median sternotomy, pericardiotomy, left pulmonary vein snaring, and pulmonary artery direct cannulation with a prototype pulmonary suffusion catheter. After occluding arterial inflow with the catheter and venous outflow with the snares, blood from the left lung was aspirated and replaced with 25% of a systemic dose of DOX diluted in 50 mL of 0.9% saline.^[^ ^32^ ^]^ For the SPME sampling, two needles were inserted into each left lobe (inferior and superior) 20 min before chemo‐suffusion, and 6 needles were inserted into 3 sections (roughly defined as upper, middle, and lower sections of each lobe of the left lung) 10 min after the suffusion. After a 1‐h suffusion followed by a 10‐min blood reperfusion, the same 6 spots were sampled again for 20 min. The SPME probes were then cleaned and analyzed as previously described.

### Animal and Human Studies—In Vivo Sampling During Localized Interstitial Chemo Phototherapy in Woodchuck Liver

A woodchuck with locally advanced hepatocellular carcinoma (HCC) tumors was treated with 2 mg kg^−1^ PhotoDOX (POP Biotechnologies, Inc., Buffalo, NY) followed by interstitial light treatment.^[^ ^33^ ^]^ PhotoDox was a phospholipid liposome incorporating a photosensitive drug in its phospholipid bilayer, loaded with DOX. Upon light activation, the encapsulated DOX was released into the tumor tissue, a targeted therapy referred to as interstitial chemo phototherapy (I‐CPT). In this study, SPME sampling was conducted post PhotoDox injection, but prior to light activation and immediately following light activation. During treatment and SPME sampling, the woodchuck was anesthetized with isoflurane mixed with oxygen at 5% for induction and 2‐3% for maintenance. The woodchuck was placed on a warm water circulation pad and prepped for surgery by removing hair from the abdomen and anterior chest and disinfecting the skin with chlorhexidine, betadine, and alcohol. A ventral midline skin incision starting at the xiphoid and extending 1–2 inches was made. The abdomen was opened via the midline incision to expose the tumor and surrounding normal liver. The SPME needles were inserted into the tumor and surrounding normal liver for 20 min and then removed. For I‐CPT, light was delivered to the target tumor through one or more light diffusing optical fibers inserted through sterilized plastic 16G catheters placed directly into the tumor tissue. Light treatment was administered for up to 2 h. Immediately following light treatment, SPME sampling was conducted by inserting the SPME needles into the tumor for 20 min.

### Animal and Human Studies—In vivo Sampling During In Vivo Lung Perfusion for Human

The surgical procedure for the open‐chest operation was described elsewhere.^[^ ^34^ ^]^ Once pulmonary and arterial bypasses were established, IVLP of the patient's left lung was performed with STEEN^tm^ perfusate containing DOX at 7 mg mL^−1^ or OxPt at 20 mg mL^−1^. For DOX, the perfusion time was 3 h, and for OxPt, the perfusion time was 2.5 h. The SPME probes were inserted into each lobe of the lung ≈30 min before IVLP (baseline), 1 h after IVLP, 2 h after IVLP, 2.5 or 3 h after IVLP, and 30 min after blood reperfusion. In some human cases, only 1 needle was used before perfusion and after reperfusion. After sampling, each probe was briefly cleaned with a Kimwipe to remove blood, rinsed in ultrapure water for 5 s using a benchtop vortex mixer, gently dried with a Kimwipe, then stored in an amber glass vial on dry ice (later transferred to ‐80°C freezer) until analysis by LC‐MS.

### Animal and Human Studies—Ethical Statements of Animal and Human Studies

For the sampling during chemo‐suffusion of pig lung, the protocols were approved by the Institute Animal Care and Use Committee (IACUC) at Roswell Park Comprehensive Cancer Center; the project number was 1470S. For the woodchuck study, the protocols were approved by the Institutional Animal Care and Use Committee at Roswell Park Comprehensive Cancer Center; the project number was 1064W. For an in vivo human study treated by doxorubicin, this was a Phase I, non‐randomized, dose‐escalation clinical trial (NCT02811523), approved by the Institutional Review Board of Toronto General Hospital Research Institute, University Health Network on Sept 11, 2015, study number 15‐8820‐C. For the in vivo human study treated by oxaliplatin, this was a Phase I, non‐randomized, dose‐escalation clinical trial (NCT05611034), approved by the Institutional Review Board of Toronto General Hospital Research Institute, University Health Network on Sept 11, 2015, study number 20‐6152. The patients’ information is listed in Table S3 (Supporting Information).

### Patient Information and Description of Ethical Permission

The in vivo human studies are from two Phase I, non‐randomized, dose escalation studies, approved respectively by the institutional review board on Sept 11, 2015, study number 15‐8820‐C (sarcoma study) and on May 3, 2021, study number 20‐6152 (colorectal study) in Toronto General Hospital Research Institute. The patents information was shown in Supporting Information.

## Conflict of Interest

Janusz Pawliszyn, Runshan Will Jiang and Wei Zhou hold a US patent application (US 2023/0273195 A1) for the related acupuncture needles with recessed coating and the push‐pull SPME microsyringe. Gal Shafirstein acknowledges the following potential conflict of interest within the submitted work: POP Biotechnologies, Inc. NCI/NIH award number R42CA243954 for his service as the PI of the subaward at Roswell Park. He is co‐inventor in US Patent 11040217, as part of his employment at Roswell Park that owns all the intellectual properties in this technology. E. Gawrys acknowledges being a co‐inventor in PCT US20230173301 as part of her employment at Roswell Park, used in the submitted work.

## Supporting information



Supporting Information

## Data Availability

The data that support the findings of this study are available from the corresponding author upon reasonable request.
