# Supplementary material for: Minimally Invasive Chemical Biopsy Needle with Self‐Wettable Extraction Phase For In Vivo Tissue Sampling During Medical Procedures
Source: Adv Sci (Weinh). 2025 Jul 22;13(1):e00396. doi: 10.1002/advs.202500396 (PMC12767110; doi:10.1002/advs.202500396)
Supplement: Supplementary file 1 — Supporting Information [file ADVS-13-e00396-s001.docx]

**Supporting Information**

Minimally invasive chemical biopsy needle with self-wettable extraction phase for in vivo tissue sampling during medical procedures

*Runshan Will Jiang†, Wei Zhou†, Marcelo Cypel, Todd L. Demmy, Gal Shafirstein, Guillermo Garza, Emily Gawrys, Joanna Bogusiewicz, Barbara Bojko, Janusz Pawliszyn**

**TABLE OF CONTENTS**

**Figure S1-S9**……………………………………………………………….……………………….**S3-S6**

**Table S1-S3**………………………………………………………………..………………………..**S7-S8**

**References**………………………………………………………………..………………………....**S8**

**
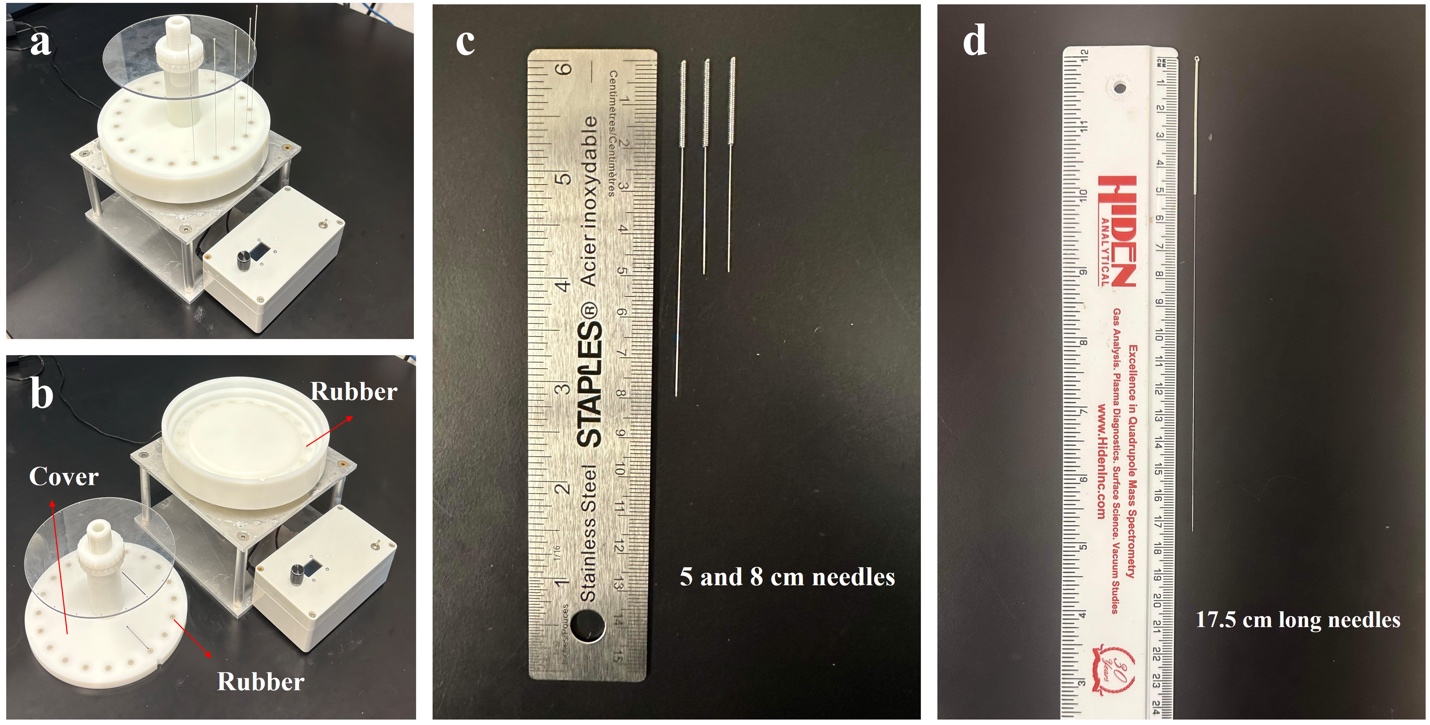
**
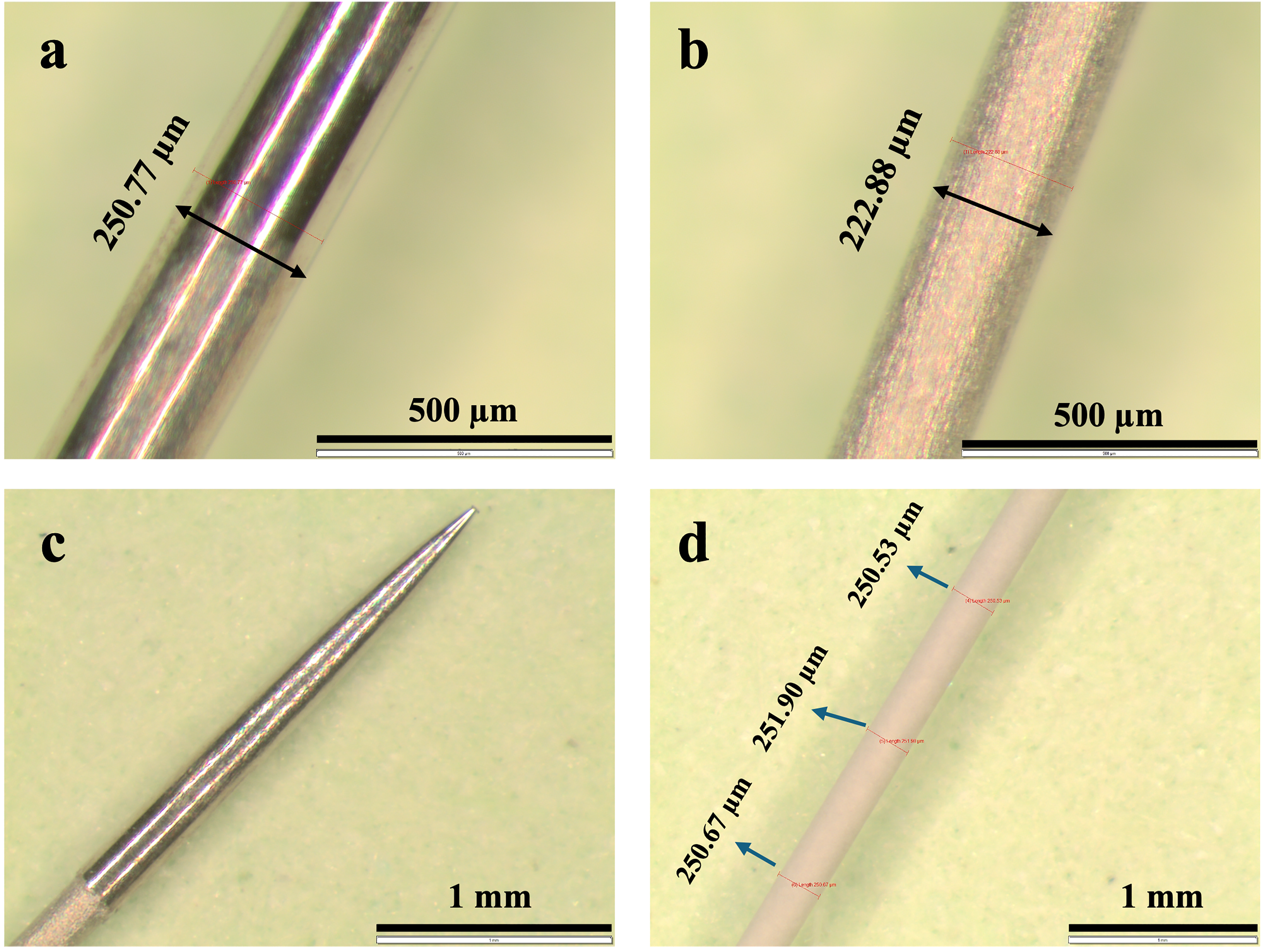
***Figure S1.*** Microscope images of the acupuncture needles. (a) Non-etching part of the acupuncture needle; (b) Recessed section after chemical etching; (c) The needle without chemical etching and (d) The recessed section with coating material.

***Figure S2.*** (a, b) Chemical etching station for acupuncture needles. (c) 5 cm and 8 cm needles with bio-compatible coating material. (d) 17.5 cm needle with bio-compatible coating material.

***
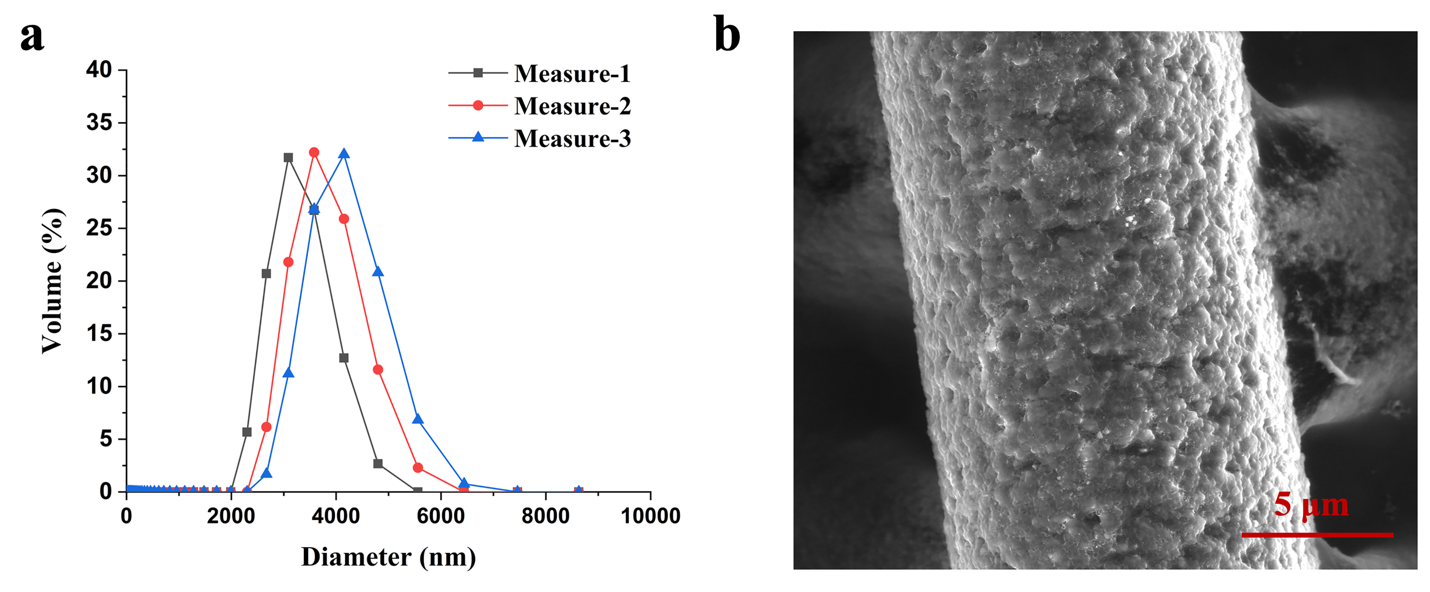
***

***Figure S3.*** (a) DLS testing of the wHLB particles; (b) SEM image of the wHLB/PAN coating.

***
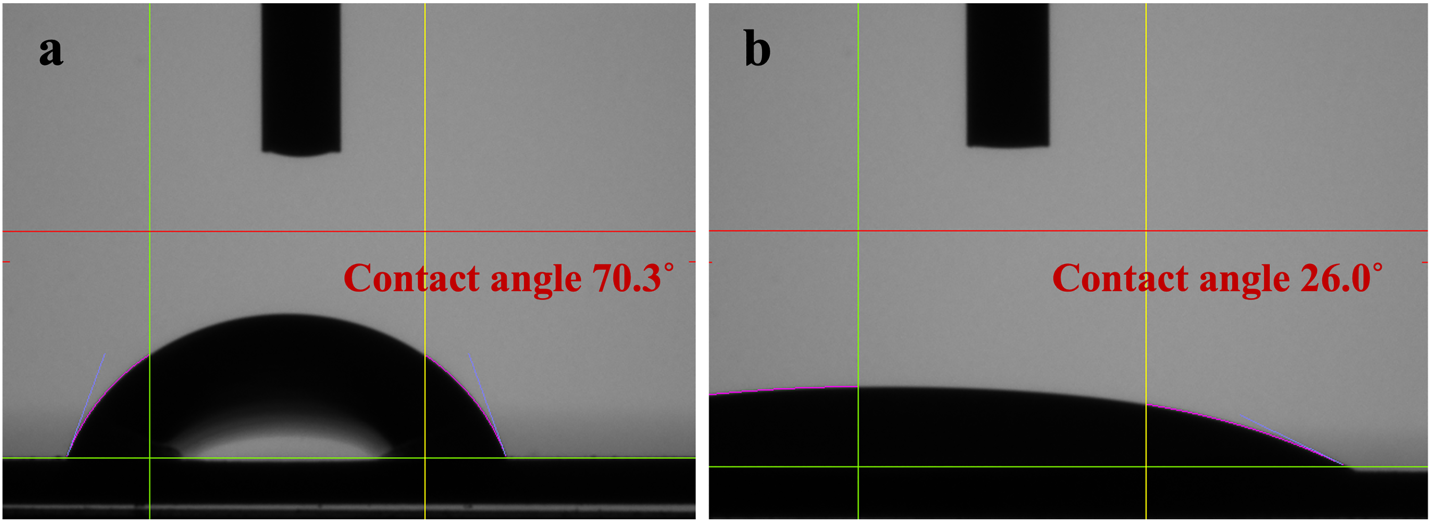
***

***Figure S4.*** Contact angle test of (a) regular HLB/PAN coating and (b) wHLB/PAN coating.

***
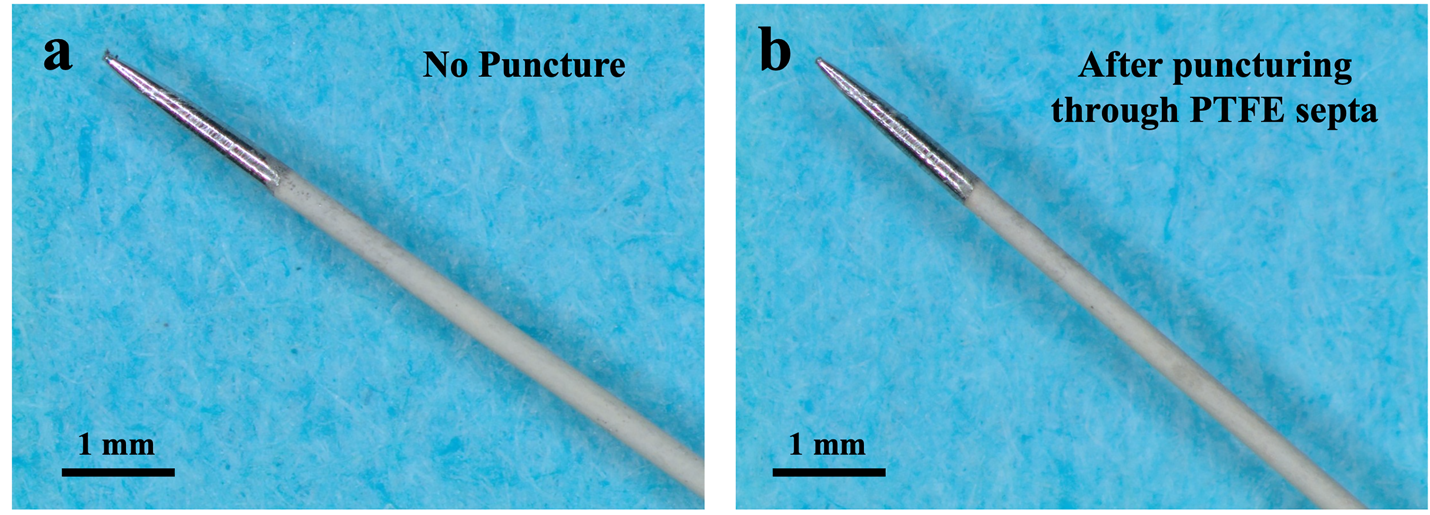
***

***Figure S5.*** Microscope images of the coated acupuncture needle (a) before and (b) after puncturing through the PTFE septa (3.5 mm in thickness for GC inlet).

***
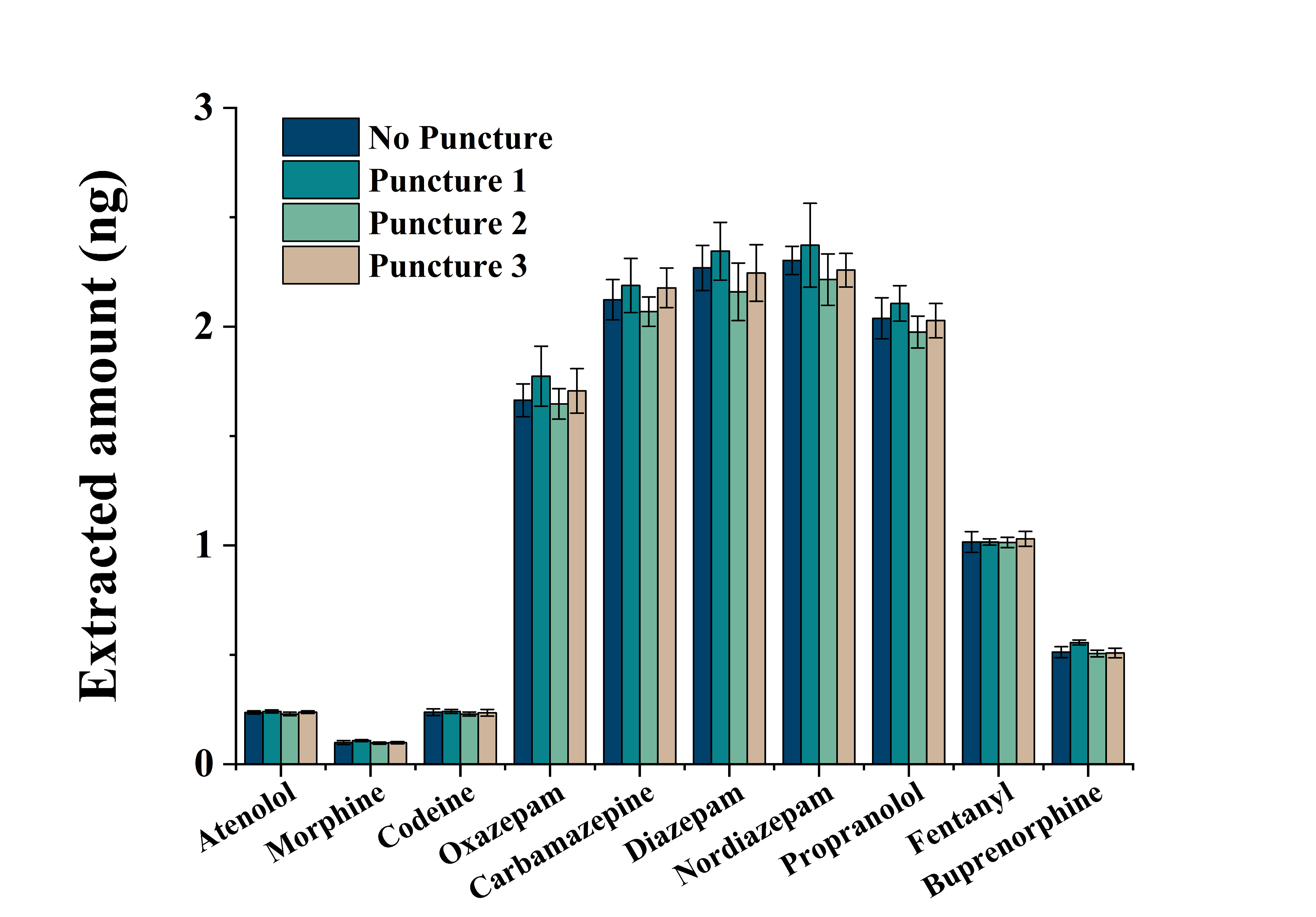
Figure S6.*** Comparison of the extraction performance of the coated acupuncture needle before and after puncture (Puncture 1 refers to puncture through PTFE septa, Puncture 2 refers to puncture through pig heart and Puncture 3 refers to puncture through pig liver).

***
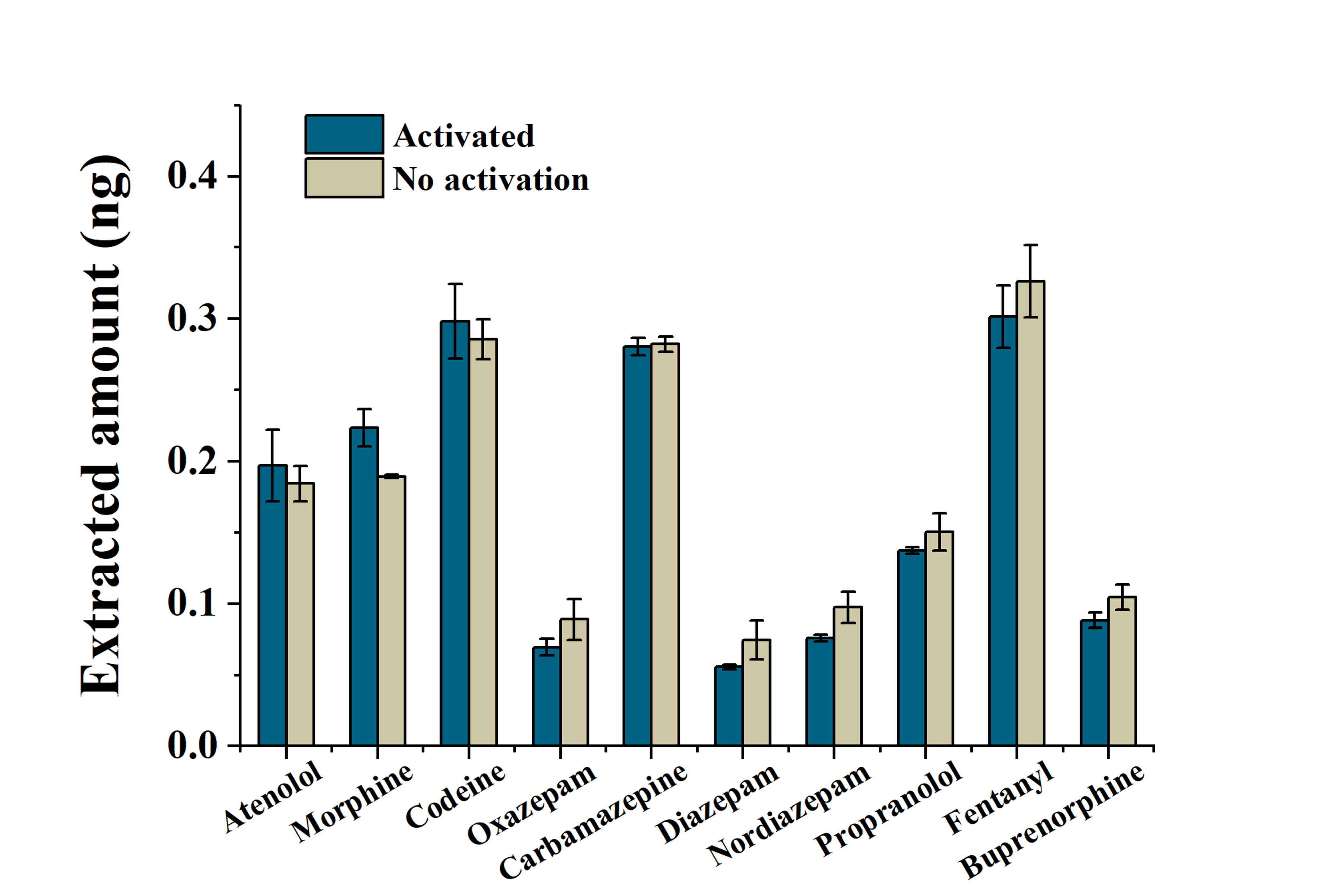
Figure S7.*** Extraction performance of wHLB/PAN coating with and without activation for the analysis of 10 drugs in human blood samples.


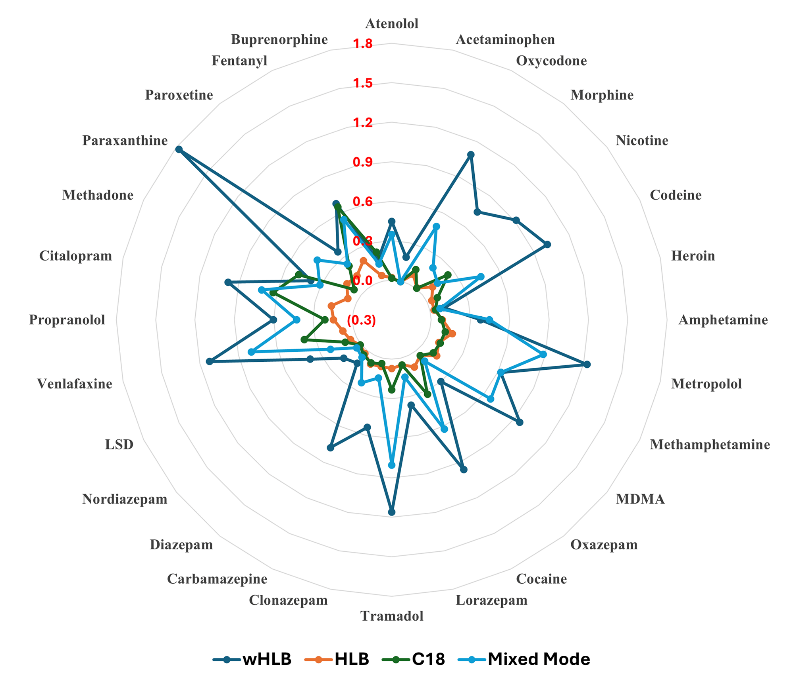


**
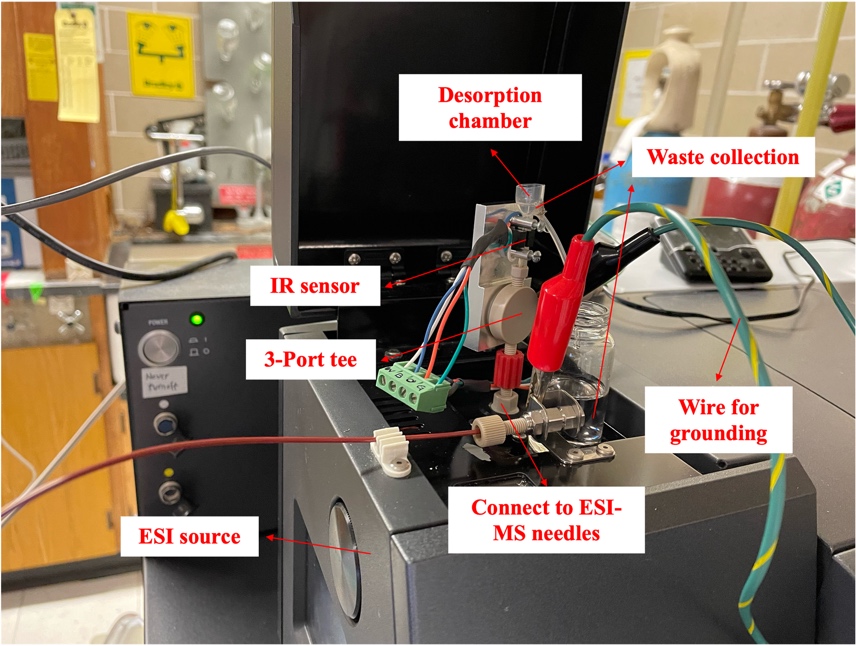
*Figure S8.*** Comparison of the extraction coverage and efficiency of the wHLB/PAN coating with commercial C18, mixed-mode and HLB/PAN coatings. 28 drugs with wide log P range were pre-spiked into the human plasma and used for testing. Data points represent the average of four technical replicates.

***Figure S9.*** The photo of the automated MOI setup on a commercial ESI-MS interface.

**Table S1.** Diameter of the recessed section of acupuncture needles after chemical etching.

| Needle number | Batch 1 | Batch 2 | Batch 3 |
| --- | --- | --- | --- |
| 1 | 220 | 219 | 221 |
| 2 | 220 | 217 | 219 |
| 3 | 217 | 217 | 223 |
| 4 | 220 | 215 | 220 |
| 5 | 219 | 217 | 220 |
| 6 | 220 | 219 | 221 |
| 7 | 219 | 215 | 215 |
| 8 | 230 | 220 | 221 |
| 9 | 215 | 220 | 229 |
| 10 | 219 | 217 | 223 |
| 11 | 220 | 219 | 221 |
| 12 | 220 | 217 | 219 |
| 13 | 218 | 217 | 221 |
| 14 | 217 | 215 | 227 |
| 15 | 220 | 210 | 220 |
| 16 | 217 | 217 | 221 |
| 17 | 217 | 219 | 221 |
| 18 | 223 | 215 | 221 |
| 19 | 220 | 219 | 217 |
| 20 | 219 | 217 | 220 |

Unit of the diameter is μm.

**Table S2.** Elemental Analysis (EA) of wHLB particles.

| Particle | Total elemental | | | |
| --- | --- | --- | --- | --- |
|  | %C | ±%C | %N | ±%N |
| Regular HLB^1^ | 89.1 | 0.3 | 0.92 | 0.03 |
| 1:2 NVP:DVB | 87.5 | 0.2 | 0.87 | 0.01 |
| 1:1 NVP:DVB | 88.4 | 0.3 | 1.13 | 0.01 |
| 2:1 NVP:DVB | 87.6 | 0.1 | 1.34 | 0.01 |
| 3:1 NVP:DVB | 86.7 | 0.2 | 1.59 | 0.02 |
| 4:1 NVP:DVB | 86.7 | 0.1 | 1.76 | 0.01 |
| 5:1 NVP:DVB | 84.7 | 0.2 | 2.59 | 0.01 |

^1^Regular HLB is synthesized according to our previous work.^[1]^

**Table S3.** Patients’ information related to human clinical trial studies.

Type: Sarcoma-Treated with IVLP of DOX, clinical trial number: NCT02811523.

| **Patient** | **Gender** | **Age** | **Recurrence** | **Death** |
| --- | --- | --- | --- | --- |
| **1** | M | 24 | N | N |

Type: colorectal cancer metastases-Treated with IVLP of OxPt, clinical trial number: NCT05611034.

| **Patient** | **Gender** | **Age** | **Recurrence (treated lung)** | **Death** |
| --- | --- | --- | --- | --- |
| **1** | M | 34 | Y | N |
| **2** | M | 51 | N | N |
| **3** | M | 59 | Y | N |
| **4** | F | 39 | N | N |

**Reference**

[1] J. J. Grandy, V. Singh, M. Lashgari, M. Gauthier, J. Pawliszyn, *Anal. Chem.* **2018**, *90*, 14072-14080.
